# Peptides for Health Benefits 2020

**DOI:** 10.3390/ijms23126699

**Published:** 2022-06-16

**Authors:** Cristina Martínez-Villaluenga, Blanca Hernández-Ledesma

**Affiliations:** 1 Institute of Food Science, Technology and Nutrition (ICTAN-CSIC), Jose Antonio Novais 10, 28040 Madrid, Spain; c.m.villaluenga@csic.es; 2 Institute of Food Science Research (CIAL, CSIC-UAM, CEI UAM + CSIC), Nicolás Cabrera 9, 28049 Madrid, Spain

In recent years, peptides have received increased interest in pharmaceutical, food, cosmetics, and various other fields. The high potency, specificity, and good safety profile are the main strengths of bioactive peptides as new and promising therapies that may fill the gap between small molecules and protein drugs. Peptides possess favorable tissue penetration and the capability to engage in specific and high-affinity interactions with endogenous receptors. The positive attributes of peptides have driven research in evaluating peptides as versatile tools for drug discovery and delivery. In addition, among bioactive peptides, those released from food protein sources have acquired importance as active components in functional foods and nutraceuticals because they are known to possess regulatory functions that can lead to health benefits.

This Special Issue of International Journal of Molecular Sciences represents the third in a series dedicated to peptides. This issue includes thirty outstanding papers describing examples of the most recent advances in peptide research and its applicability. 

The Special Issue starts with a group of papers investigating the potential of synthetic peptides as new drug alternatives for controlling and/or managing chronic diseases. It begins with a study by Lee et al. [1] on the evaluation of the biochemical and biological properties of PEG-BHD1028, a novel peptide agonist to adiponectin receptors, by in vitro and in vivo testing models. These findings will open interesting perspectives to this peptide and its analogs’ applicability as potential adiponectin replacement therapeutic agents. The issue follows with the research of Shah & Chen [2] on the antimicrobial peptide Sub5, a synthetic variant of bactenecin 2A with substitutions of five amino acids of a total of 12 amino acid sequence. *Saccharomyces cerevisiae* proteome microarrays are employed to discover the direct protein targets of Sub5 from Sub5-protein interactions. Bioinformatics analysis reveals 15 actin-associated proteins as targets of Sub5. The protein–protein interaction network is linked to ribonucleoprotein, transcription and translation, chromosome, histone, and ubiquitin-related DNA repair, and chaperone. Ramos-Martin and D’Amelio [3] elucidate, by molecular dynamics simulations and sequence-property alignment, the mechanism of action of cecropinXJ, a cationic antimicrobial peptide belonging to the cecropin-B family and isolated from larvae of *Bombyx mori*. Besides its potent activity against several highly virulent and antibiotic resistant bacterial pathogens, cecropin KJ has demonstrated to be one of the few peptides active against esophageal cancer. In the study of Bryzek et al. [4], arginine residues in human cathelicidin LL-37 are substituted by homoarginine citrulline to produce a new generation of agents to treat bacterial infections and other inflammatory diseases associated with peptidyl-arginine deiminases activity. Binder and Skerra [5] combine PASylation^®^ technology with enzymatic in situ N-acetylation by RimJ to obtain a long-acting version of immunostimulatory peptide thymosin α1 in *Escherichia coli* at high yield. The findings of this study provide the basis for the therapeutic development of a next generation thymosin α1 with prolonged plasma circulation that promotes its benefits against hepatitis B and C viruses, among other infections. The study of Colvin et al. [6] investigates the impact of Ex-4, a glucagon-like peptide-1 (GLP-1) analogue, on the rewarding properties of palatable food and alcohol consumption when administered to multiple brain areas involved in mesocorticolimbic reward circuitry and homeostatic mechanisms associated with motivation. Novel regions of the central nervous systems, including the dorsomedial hippocampus and lateral hypothalamus, are identified, opening new therapeutic interventions acting on these regions to mediate drug reward. The Special Issue also contains the study of Bartlett et al. [7] on BBI-11008, a glycopeptide analog of frog skin-derived deltorphin. The finding of this study illustrates an example of significant blood–brain barrier penetration of the glycopeptide analogue to achieve a central behavioral effect, providing additional evidence for the glycosylation technique as a method to harness therapeutic potential of peptides. The research of Kim et al. [8] demonstrates that *Toxoplasma gondii*-secreted dense granule protein (GRA9) interact with recognition receptor NLRP3 and inhibit the formation of the NLRP3 inflammasome through the blockade of the binding of apoptotic speck-containing (ASC) to NLRP3 in mitochondria. In an *E. coli*- or *P. aeruginosa*-induced sepsis model mice, recombinant GRA9C increase the anti-inflammatory, bactericidal, and anti-septic effects by increasing M2 polarization. These findings define the potential of GRA9 as a new candidate to be a therapeutic agent for sepsis. Kanellopoulos et al. [9] report the impact of registered neprilysin (NEP)/angiotensin-converting enzyme (ACE) inhibitors on the performance of [99mTc]Tc–DT1 and [99mTc]Tc–DT5 in both pancreatic cell culture and xenograft cancer animal models. In another paper, Korokin et al. [10] use a rat model of preeclampsia, known as a severe disease of late pregnancy, to demonstrate the endothelial dysfunction correcting properties of erythropoietin mimetic peptide (pHBSP). Apart from the reduction of systolic and diastolic blood pressure, pHBSP decreases the coefficient of endothelial dysfunction, increases the placental microcirculation and NOx concentration, diminishes the greater momentum edema and proteinuria, and improves the morphological pattern of the fetoplacental complex and the ratio of BAX to Bcl-2 expression, which characterizes the apoptotic orientation of the cells. In the study of Nasako et al. [11], peptide PMTPV, which mimics the structure of second extracellular loop of tight junctional protein claudin-1, is presented as a potential chemosensitizer for lung adenocarcinoma after elucidating its mechanism action in both parental and cisplatin-resistance A549 cells. Then, in the study of Insuasty-Cepeda et al. [12], the substitution of ^26^Met by hydrophobic amino acids in the sequence of bovine lactoferricin-derived dimeric peptide LfcinB results in a significant enhancement of its cytotoxic activity against breast cancer HTB-132 and MCF-7 cells without affecting non-tumorigenic MCF-12 cells. Moreover, the obtained dimeric peptides promote apoptosis through the intrinsic pathway and do not compromise the integrity of the cytoplasmic membrane. These findings show the promising role of these molecules as basis for the design and development of new drugs against breast cancer. Finally, Wilson et al. [13] assess the T cell response to different vaccine delivery systems by using model peptide SYIPSAEKI epitopes that have been systematically altered by changing each position along the peptide outside the T cell recognition and major histocompatibility complex anchor sites (^2^Tyr and ^9^Ile) to an alanine (SYIPSAEAI, SYIPSAAKI, SYIPAAEKI, SYIASAEKI, SYAPSAEKI, AYIPSAEKI). The findings of this study offer new strategies for the design of more efficient peptide-based vaccines to achieve a desired immune response.

Moreover, there is a short series of articles dealing with research in food-derived bioactive peptides, including the characterization of chemical structure and elucidation of modes of action of food-derived bioactive peptides. The review of Jahandideh & Wu [14] discusses the role played by the renin-angiotensin system (RAS) on the pathogenesis of the metabolic syndrome and summarizes the existing in vitro and in vivo evidence on the RAS-modulating effects of food bioactive peptides. In silico and in vitro assays are conducted by Iwaniak et al. [15] to evaluate the angiotensin-converting enzyme (ACE) and dipeptidyl peptidase (DPP-IV) enzyme of Gouda cheese with a modified content of β-casein. Different Gouda cheeses with decreased, increased, and normal content of β-casein after 1 and 60 days of ripening each are analyzed, observing that all variants exhibit comparable ACE inhibition, whereas DPP-IV inhibition is more diversified among the samples. Furthermore, the research of Fan et al. [16] clonee the soybean-derived lunasin into pCAMBIA3300 and transferred the expression vector into wheat via an *Agrobacterium*-mediated transformation. Lunasin is not detected in wild-type wheat while it is present in transgenic wheat. Moreover, lunasin enrichment from transgenic wheat displays an increased anti-proliferative activity in HT-29 cells through induction of pro-apoptotic pathways. The review of Tyagi et al. [17] discusses current knowledge on opioid endogenous and exogenous peptides focusing on their sources, production, purification, impact on stress, anxiety, and depression, and mechanisms of action. The Special Issue proceeds with the review of Tulipano [18] about the role of bioactive peptide sequences in the potential impact of dairy protein intake on metabolic health. The articles summarized by this review suggest the beneficial effects exerted at local and/or systemic level of peptides released from milk proteins during their transit through the gastrointestinal tract and describe the relevance of the enteroendocrine system in the cross talk between food proteins and the neuroendocrine network regulating energy balance. Finally, the study of Lutaty et al. [19] on two bioactive peptides, FKD and FKE, contained in lactoferrin sequence, report their ability to modulate human macrophages reprogramming to an anti-inflammatory/pro-resolving phenotype through the inhibition of lipopolysaccharide (LPS)-induced TNF-α and IL-6 secretion and an increase in IL-10 levels.

The Special Issue includes some studies on identification and assessment of biological activity of natural peptides produced by animals and microganisms. El Aziz et al. [20] screen the venom of the Egyptian black snake, *Walterinnesia aegyptia*, for bioactive compounds. Walterospermin, a peptide of 57 amino acid residues with high homology to other venom toxins, was identified. Moreover, its ability to activate sperm motility from a variety of species (including humans) through the interaction with the receptor that controls motility function was proven. De Waard et al. [21] explore the biological effects of BeKm-1, a natural scorpion venom peptide. By delaying cardiomyocyte repolarization, BeKm-1 induces early afterdepolarizations and reduces spontaneous action potentials, calcium transients, and contraction frequencies. These critical phenotype features are associated with drug-induced long QT syndrome, a life-threatening disorder characterized by ventricular arrhythmias. Being a peptide that is easily modifiable, BeKm-1 will serve as an ideal molecular platform for the design of new hERG modulators displaying additional functionalities. Antimicrobial peptides are naturally and ubiquitously synthesized by microbes, plants, animals, and humans as a first-line host defense with potent anti-bacterial, anti-virus, anti-fungal, and anti-cancer activities. Raileanu et al. [22] evaluated the efficiency of the antimicrobial peptide gramicidin A and the anticancer drug, doxorubicin, against the spheroids from colorectal cancer cells (HT-29). The results show that simultaneous use of the two compounds cause a synergistic effect on reduction of cell viability and cellular ATP levels. Liu et al. [23] review major common foodborne pathogens and their huge impact on human health as well as on the food industry. Next, the review describes persisting morphotypes of the mentioned five foodborne pathogens and the mechanisms of their formation. Following that, it summarizes recent progress made in research on antimicrobial peptides, and it describes their identified modes of action and their application in persister eradication, food preservation, and pathogen detection. Finally, we provide a brief overview of the current challenges for antimicrobial peptides to become anti-persister agents of the future.

Another group of papers explores the effects of human endogenous peptides on body functions. Retinal aging is the result of accumulating molecular and cellular damage with a manifest decline in visual functions. Pöstyéni et al. [24] validate a transgenic model for somatostatin-expressing amacrine cells and investigate the chronic effect of pituitary adenylate cyclase-activating polypeptide on the aging of somatostatinergic and dopaminergic cells of the retina. The novelty herein is that pituitary adenylate cyclase-activating polypeptide could increase the cell density in matured retinal tissue, anticipating new therapeutic potential in age-related pathological processes. Golovin et al. [25] focuses on the study of transactive response DNA and RNA binding protein 43 kDa (TDP-43), a nuclear ribonucleoprotein playing a role in all steps of mRNA life cycle that is associated with several neurodegenerative diseases. This study suggests the existence of a zinc binding site in the C-terminal region of RRM2 domain of TDP-43 and proposes a model of its complex with Zn^2+^, which illustrates how zinc might regulate DNA/RNA. Wojciechowski et al. [26] examine the potency of neuropeptide FF to block post-opioid respiratory depression, one of the main adverse effects of opioid therapy. Main findings of this study point out that centrally administered neuropeptide FF is effective in preventing apnea evoked by stimulation of μ-opioid receptors, and the effect was due to activation of central neuropeptide FF receptors. These findings indicate a potential target for reversal of opioid-induced respiratory depression. Hó et al. [27] describes human leukocyte antigen (HLA)-Ib presentation of hemoglobin-derived peptides in CD4+ T-cells, a known mediator of HIV progression. Peptide sequence analysis from HLA- Ib allelic variants featured hemoglobin peptides as dominant and shared. The reciprocal experiment of binding hemoglobin peptide fractions to the HLA-Ib open conformers resulted in significantly diminished receptor recognition. These results underpin the molecular involvement of HLA-F and its designated peptide ligand in HIV immune escape. Billert et al. [28] review the current knowledge in pleiotropic effects of phoenixin neuropeptide, a cleaved product of the Smim20 protein. Data indicate that the biological effects of PNX in reproduction, behavior, memory, sensory processes, fluid homeostasis, food intake, and glucose as well as in lipid metabolism are mediated through the GPR173 receptor. Pandey et al. [29] confirmed the presence of neuropeptide B/W signaling system in heart, DRG, and stellate ganglia by proteomic and genomic analyses. This signaling system has a wide spectrum of functions including a role in modulation of inflammatory pain and neuroendocrine functions.

Finally, an article describes newly developed biomaterials based on peptides for biomedical applications. Antifouling polymer layers containing extracellular matrix-derived peptide motifs offer promising new options for biomimetic surface engineering. Sivkova et al. [30] report the design of antifouling vascular grafts bearing biofunctional peptide motifs for tissue regeneration applications based on hierarchical polymer brushes. The hierarchical brushes presenting the RGD sequences provided excellent cell adhesion properties and at the same time remained resistant to fouling from blood plasma. The obtained bioactive antifouling vascular grafts promoted the specific adhesion and growth of endothelial cells, thus providing a potential avenue for endothelialization of artificial conduits.

We wish to thank the invited authors for their interesting and insightful contributions, and look forward to a new set of advances in the bioactive peptides field to be included in the following Special Issue, “Peptides for Health Benefits 2021”, (https://www.mdpi.com/journal/ijms/special_issues/peptides_2021, accessed on 20 May 2022).

## Data Availability

Not applicable.
